# 3D Bioprinting of Human Adipose-Derived Stem Cells and Their Tenogenic Differentiation in Clinical-Grade Medium

**DOI:** 10.3390/ijms21228694

**Published:** 2020-11-18

**Authors:** Deborah Stanco, Monica Boffito, Alessia Bogni, Luca Puricelli, Josefa Barrero, Gianni Soldati, Gianluca Ciardelli

**Affiliations:** 1 Department of Mechanical and Aerospace Engineering, Politecnico di Torino, Corso Duca degli Abruzzi 24, 10129 Turin, Italy; deborah.stanco@polito.it (D.S.); monica.boffito@polito.it (M.B.); gianluca.ciardelli@polito.it (G.C.); 2 European Commission, Joint Research Centre (JRC), Via E. Fermi, 2749, 21027 Ispra, Italy; alessia.bogni@ec.europa.eu (A.B.); luca.puricelli@ec.europa.eu (L.P.); 3 Swiss Stem Cell Foundation, Via in Pasquée 32, 6925 Gentilino, Switzerland; gianni.soldati@gmail.com

**Keywords:** 3D bioprinting, adipose-derived stem cells, tendon tissue engineering, tenogenic differentiation, xenogenic-free, collagen, scleraxis, nanocellulose, alginate

## Abstract

Defining the best combination of cells and biomaterials is a key challenge for the development of tendon tissue engineering (TE) strategies. Adipose-derived stem cells (ASCs) are ideal candidates for this purpose. In addition, controlled cell-based products adherent to good manufacturing practice (GMP) are required for their clinical scale-up. With this aim, in this study, ASC 3D bioprinting and GMP-compliant tenogenic differentiation were investigated. In detail, primary human ASCs were embedded within a nanofibrillar-cellulose/alginate bioink and 3D-bioprinted into multi-layered square-grid matrices. Bioink viscoelastic properties and scaffold ultrastructural morphology were analyzed by rheology and scanning electron microscopy (SEM). The optimal cell concentration for printing among 3, 6 and 9 × 10^6^ ASC/mL was evaluated in terms of cell viability. ASC morphology was characterized by SEM and F-actin immunostaining. Tenogenic differentiation ability was then evaluated in terms of cell viability, morphology and expression of scleraxis and collagen type III by biochemical induction using BMP-12, TGF-β3, CTGF and ascorbic acid supplementation (TENO). Pro-inflammatory cytokine release was also assessed. Bioprinted ASCs showed high viability and survival and exhibited a tenocyte-like phenotype after biochemical induction, with no inflammatory response to the bioink. In conclusion, we report a first proof of concept for the clinical scale-up of ASC 3D bioprinting for tendon TE.

## 1. Introduction

The inability of native tendons to de novo synthesize their extracellular matrix (ECM) and restore their functions after injury is expected to be overcome by cell-based tissue engineering. Tissue engineering (TE) strategies aim to deliver adequate, regeneration-competent cells, biomaterials and signaling factors to the injured tendon site [1]. In particular, cells play a primary role in the development and maintenance of tendon extracellular matrix homeostasis; biomaterials properly micro-fabricated into scaffolds could mimic the natural microenvironment to provide mechanical-physical support and a differentiation microenvironment. Furthermore, the in situ secretion of signaling factors as growth factors could progressively guide tissue development. Mesenchymal stem cells (MSCs) are localized in almost all adult tissues, representing the ideal cell source for tissue engineering strategies [2,3,4,5,6]. Although tendon-derived MSCs could be used as an autologous cell source for tendon reconstruction, the low cellular yields and the potential donor side morbidity have limited their potential application in this field [7,8]. Adipose-derived stem cells (ASCs) have been intensively studied in recent decades for their self-renewal and regenerative ability. They are present in abundancy in white adipose tissue, and the easy and minimally invasive procedure for their harvest makes them ideal for application in cell-based therapies [8,9,10]. Moreover, it is known that ASCs can release a broad panel of growth factors and cytokines with trophic, immunomodulatory, antiapoptotic and proangiogenic properties, such as the hepatocyte growth factor (HGF), granulocyte and macrophage colony-stimulating factors (GM-CSF), interleukins (ILs) 6, 7, 8 and 11, tumor necrosis factor-alpha (TNF-α), vascular endothelial growth factor (VEGF), brain-derived neurotrophic factor (BDNF), nerve growth factor (NGF), adipokines and others [3]. For these reasons, ASCs have the potential to be employed for tendon TE as they are an efficient and safe tool to modulate the tendon niche microenvironment, reduce inflammation and improve tendon healing in vivo [11,12,13]. Although the optimal conditions to drive tendon cell differentiation have not been identified yet, several attempts have been made in vitro to induce ASC tenogenic differentiation using growth factors [5,8,14,15], mechanical cues [16] or a combination of both stimuli [17]. We have previously developed a tenogenic protocol to trigger ASC differentiation toward the tendon lineage in vitro by supplementing the culture medium with growth factors and molecules (hereafter referred to with the abbreviation TENO) highly expressed during the early phase of tendon development and the tendon healing process, such as bone morphogenic protein-12 (BMP-12), transforming growth factor-beta3 (TGF-β3), connective tissue growth factor (CTGF) and ascorbic acid [5]. Importantly, in order to move towards the clinical application of a safe and standardized ASC-based product adherent to good manufacturing practice (GMP), clinical-grade reagents were selected as TENO medium constituents, thus avoiding the use of products derived from animal sources (i.e., fetal bovine serum—FBS) [18]. We demonstrated that ASCs cultured in TENO xenogenic-free medium exhibited a tenocyte-like phenotype by expression of specific tendon-related markers such as scleraxis, collagen type I/III, tenomodulin, COMP and metalloproteinase-1/-3. The peculiar tendon architecture and its ECM composition are essential for tendon functionality, cell microenvironment modulation and the mechano-sensitivity and mechano-responsive properties. The 3D environment plays a key role in driving cell behavior and fate further and thus scaffolding strategies could be exploited to design suitable cell substrates for tendon TE mimicking the specific ECM composition and the multiple hierarchical collagen fibrous structure of tendon tissue [19]. In this context, nanofibrous materials have recently been studied because of their natural microporous structure resembling the ultrastructure of tendon tissue, that could provide cell–cell interactions and proper porosity to encourage remodeling [20]. For instance, Yang et al. recently reported the design of a multi-layered fiber–hydrogel scaffold to closer mimic the tendon structure by simultaneous co-electrospinning of poly(caprolactone) (PCL) and methacrylated gelatin. The combination of nanofibrous structures and hydrogels provided aligned topographical cues and contributed to the tenogenic differentiation of embedded ASCs [21]. However, conventional fabrication techniques are scarcely applicable in the clinical setting for their limited scale-up production or standardization [19]. Moreover, the traditional tissue-engineered construct production, consisting of manual cell seeding over the scaffold, possesses several issues and drawbacks, including a non-homogenous cell distribution, the inability to spatially distribute multiple cell types and the poor control over the scaffold microarchitecture. Three-dimensional (3D) bioprinting has recently emerged as a promising tool that can potentially overcome these limitations, allowing 3D complex cell culture system fabrication with precise and controlled cell positioning, which finally results in highly reproducible bioengineered cell-laden constructs [22]. The advantage of using such biomimetic 3D-printed scaffolds relies in the possibility to recapitulate key aspects of the tissue environment allowing cell–cell and cell–matrix interactions which are lacking in standard monolayer cell cultures. Among the available printing systems, the extrusion-based method allows the microfabrication of complex structures combining different cell types and materials into a bioink that can be printed with high cell densities under cell-friendly conditions [23].

To date, the literature reports only one study applying 3D bioprinting for muscle–tendon junction TE [24]. In this work, the authors fabricated a muscle–tendon unit construct composed of thermoplastic structural polymers used as a scaffold for the bioprinting of two cell-laden hydrogels that provided terminally differentiated myoblasts on the muscle part and fibroblasts at the tendon part. The functionality of the construct was proved by collagen type I deposition at the tendon side after seven days of culture in standard FBS medium.

In such a context, characterized by a scarce number of reports concerning tendon tissue engineering based on 3D bioprinting and in order to move towards a clinical scale-up of the ASC-based approach, the main aim of this study was to investigate the 3D bioprinting of primary human ASCs and to verify the in vitro functionality of the built 3D constructs in terms of cell viability and differentiation towards tenogenic lineage. The bioink used here is composed of nanofibrillar cellulose (NFC) and alginate (A) combined with a blend of ECM-laminin proteins (Laminink+, Cellink) to resemble the tendon microarchitecture and ECM composition as well as to support cellular response. Nanofibrillar cellulose and alginate are natural and xenogenic-free materials that make them particularly promising in wound healing, drug delivery and for bone and cartilage tissue engineering applications. Due to their high biocompatibility, bio-degradability, low cost and easy availability, they are widely used in the biomedical field and are already approved by the Food and drug Administration (FDA) for their use in dental applications and wound dressing [25,26,27]. In particular, nanofibrillar cellulose has been successfully employed as a reinforcing agent in many biodegradable polymeric compositions for its high tensile strength properties, capacity to retain water and tunable surface chemistry properties [28]. Moreover, its fibrillary structure resembles the collagen fibrils of a tendon. Alginate has been safely implanted in various applications for cell transplantation [29]; it forms stable gels in the presence of certain divalent cations, such as calcium ions, and provides a biocompatible environment suitable for downstream processes such as in vitro cell culture or in vivo experiments [30,31].

Only recently, few studies have been carried out to investigate the 3D bioprinting feasibility of NFC/A hydrogels for cartilage TE. In detail, the bioink turned out to allow the 3D bioprinting of the desired structures and to support proliferation and differentiation towards the chondrogenic lineage of the encapsulated chondrocytes or induced pluripotent stem cells [32,33,34,35]. Moreover, the effective in vivo functionality of the 3D-bioprinted NFC/A construct to provide new cartilage formation, structural integrity of the construct and good host tissue integration has already been proved [33]. For what concerns the safety of nanofibrillar cellulose, previous in vitro studies reported no pro-inflammatory cytokine secretion by macrophages [36,37].

To the best of our knowledge, NFC/A hydrogels have never been used as bioinks for the 3D bioprinting of ASCs and as a scaffold for their tenogenic differentiation. Most importantly, in this study, we employed already isolated ASC populations that were well characterized in terms of MSC marker expression and differentiative ability in 2D conditions, as we had demonstrated in our previous published work [5]. A suitable cell density within the bioink is essential to guarantee 3D construct functionality in terms of cell survival after printing, cell proliferation and differentiation [38]. For this reason, in order to allow the identification of the best conditions for printing and optimal cell growth, we first investigated the optimal seeding density among 3.0, 6.0 and 9.0 × 10^6^ ASCs/mL of bioink. Then, the ability of ASCs embedded in the 3D NFC/A constructs to differentiate towards the tendon lineage was assessed using our already established tenogenic-GMP-compliant protocol [5]. In detail, the tenogenic induction of 3D cell constructs was evaluated in terms of cell morphology, viability and proliferation and by monitoring the expression of the specific tendon-related markers scleraxis and collagen type III. Finally, in order to address safety and exclude unwanted negative effects depended by the NFC/A hydrogel on the biological response of undifferentiated and differentiated ASCs, the release of several pro-inflammatory cytokines in the ASCs’ secretome was also evaluated.

## 2. Results

### 2.1. Characterization of the NFC/A Hydrogel

**Figure 1:**
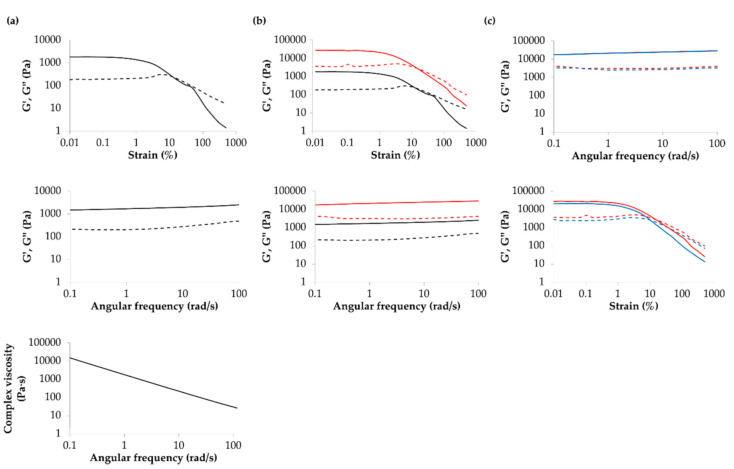
Rheological characterization of the bioink: (**a**) storage (G’, continuous line) and loss (G”, dashed line) moduli trends as a function of applied strain and angular frequency, both at 25 °C, and complex viscosity trend as a function of angular frequency for the bioink before crosslinking; (**b**) storage (G’, continuous line) and loss (G”, dashed line) moduli trends as a function of applied strain and angular frequency at 25 °C for the bioink before (black) and after (red) crosslinking; (**c**) storage (G’, continuous line) and loss (G”, dashed line) moduli trends as a function of applied strain and angular frequency at 25 (red) and 37 °C (blue) for the bioink after crosslinking.

The NFC/A bioink is a mixture of nanofibrillar cellulose and sodium alginate provided by the manufacturer in a sterile cartridge. The viscoelastic properties of the NFC/A hydrogel were rheologically analyzed at 25 °C (printing temperature). First, a strain sweep test was conducted for bioink characterization in terms of resistance to the applied deformation. Figure 1a reports the trends of storage and loss moduli (G’ and G’’, respectively) as a function of applied strain within the range 0.01–500%. The NFC/A hydrogel exhibited the typical behavior of structured materials with G’ initially constant up to a critical strain value (i.e., γ_L_ 1.8%), which identifies the limiting value of the linear viscoelastic (LVE) region. Within the LVE region, the sample exhibited the characteristic behavior of a gel system (G’ > G’’). At strain higher than γ_L_, G’ started to decrease, while G’’ initially slightly increased and then decreased, as a consequence of a slight strain hardening effect. For strain values within γ_L_ and the strain at the G’/G’’ crossover (i.e., 11.6%), micro-cracks appeared within the sample, progressively leading to the complete mechanical failure of the gel with the appearance of macro-cracks and the sample behaving as a solution (G’’ > G’). Gel yield stress resulted to be 38.7 Pa. A frequency sweep test (Figure 1a) further confirmed the gel state of the investigated sample at 25 °C. Indeed, the storage modulus trend turned out to be independent of the angular frequency within the investigated range, as is typical for fully developed gel systems. Complex viscosity linearly decreased with increasing angular frequency from 0.1 to 100 rad/s (Figure 1a), suggesting that the bioink possessed a shear thinning behavior, which is a key feature for printable formulations [39].

The same characterizations were performed also on the crosslinked gel (Figure 1b). Both strain sweep and frequency sweep tests confirmed the successful crosslinking of the gel, with a significant increase in both G’ and G’’. For instance, the G’ value at 0.01% applied strain increased from 20,200 to 26,900 Pa. The γ_L_ value slightly decreased from 1.8% to 1.1% upon crosslinking. On the other hand, yield stress increased of one order of magnitude, from 38.7 to 404 Pa. As expected, the crosslinking procedure improved the overall mechanical properties of the sample which still remained in the gel state (i.e., G’ > G’’, G’ independent of angular frequency). The crosslinked gel was further characterized at 37 °C, which will be the real working temperature of the printed constructs. Both strain sweep and frequency sweep tests evidenced the capability of the crosslinked gel to keep its mechanical properties constant, with no thermo-responsiveness (Figure 1c).

### 2.2. Characterization of the 3D NFC/A Scaffold

**Figure 2:**
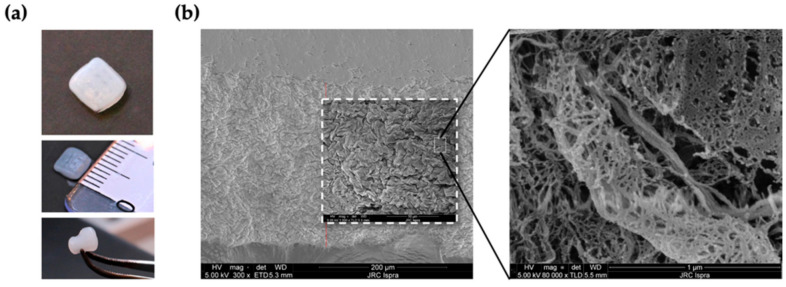
3D-printed nanofibrillar cellulose/alginate (NFC/A) scaffold: (**a**) photographs show the macroscopic characteristics and easy handling of the 3D-printed scaffolds; (**b**) scanning electron microscopy (SEM) micrographs of the 3D NFC/A hydrogel show the ultrastructural morphology of the scaffold at the top view.

The printing parameters were set in order to obtain NFC/A scaffolds with a 5 × 5 × 1 mm dimension. In order to achieve good shape fidelity of the resulting scaffolds and the preservation of cell function, the precise positioning of the bioink was enabled by a conical nozzle of 200 µm in diameter (27 gauge) and cell viability in the printed constructs was ensured by maintaining a low pressure (9–14 kPa) and speed (5 mm/sec) of deposition, which is known to minimize shear stress and thus cell damage [40]. As a result of the printing and crosslinking processes, small 3D square-shaped structures of gels were obtained, as shown in Figure 2a. Macroscopically, printed scaffolds appeared opaque, white and well defined in terms of structure with an average width of 5.72 ± 0.62 mm (measures performed with Image J software; data not shown); they could be deformed while squeezing and were easily handled using a spatula. SEM analysis was used to evaluate the microstructure and porosity of the NFC/A 3D scaffold after printing. As shown in Figure 2b, the filament surface appeared highly porous with the typical cellulose fibrillar morphology in the nanometer range (40.41 ± 35.76 nm in diameter), which plays a key role in assisting nutrient and oxygen supply to the encapsulated cells.

**Figure 3:**
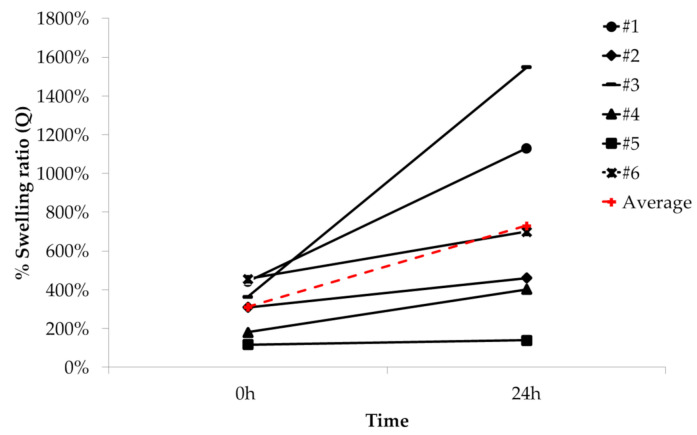
Swelling ratio (%) of each NFC/A scaffold (N = 6) and average swelling ratio at 0 and 24 h after printing.

The hydrophilicity of the scaffold was determined by measuring the swelling ratio [41]. As shown in Figure 3, the average swelling ratio of as-prepared NFC/A scaffolds after crosslinking and 10 min washing in HBSS^+/+^ at room temperature (RT) was 311.3 ± 137.8%. Then, after 24-h sample incubation in HBSS^+/+^ at 37 °C, the average swelling ratio increased (+134.3%) without statistically significant differences compared to the initial value.

### 2.3. Cell Viability and Appearance of Bioprinted ASCs

**Figure 4:**
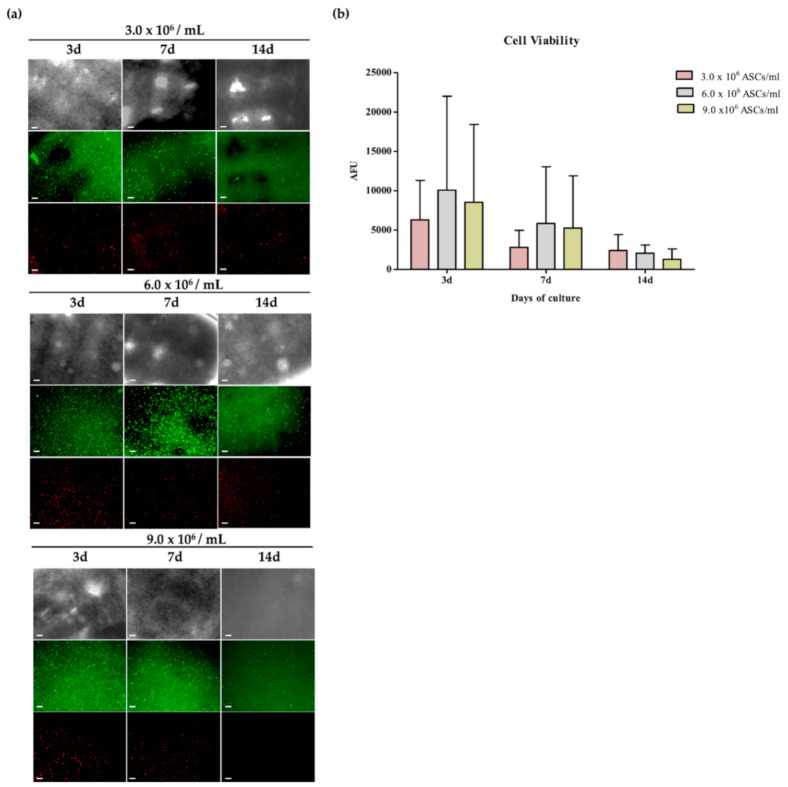
Cell viability of adipose-derived stem cells (ASCs) printed in the NFC/A hydrogel at different cell densities. (**a**) Representative images showing bright field and live and dead staining of live (green) and dead (red) encapsulated cells at 3, 6 and 9.0 × 10^6^ cells/mL of cell density captured at 3, 7 and 14 days (4× magnification; scale bar 10µm; *N* = 3); (**b**) cell viability of ASCs at 3, 7 and 14 days after bioprinting at three different cell densities (*N* = 3). Data were expressed as average ± standard deviation of arbitrary fluorescence units (AFU).

In order to identify the optimal cell density for cell survival and viability after printing, three different cell concentrations were tested (i.e., 3, 6 and 9.0 × 10^6^ ASCs per mL of bioink) at 1, 7 and 14 days after bioprinting. Live and dead staining, shown in Figure 4a, resulted as similar among the different seeding densities. In particular, cells appeared rounded and showed a very high cell viability at all time points of culture after bioprinting, as highlighted by the higher number of green cells visualized in the cell-laden constructs in comparison with the fewer red cells. No significant difference due to cell concentration in the starting bioink was observed. The same trend was maintained also after 7 days of culture, irrespective of the initial cell density within the bioink, showing a high number of living cells and only some dead cells within the 3D cell constructs. On the other hand, at 14 days, although cell viability remained significantly high, it slightly decreased in comparison with earlier time points in all printing conditions.

According to these results, cell metabolic activity evaluations confirmed the high cell viability in all 3D cell constructs already three days after bioprinting without any difference ascribable to the initial cell concentration in the bioink (Figure 4b). In particular, ASCs printed at the cell density of 6.0 × 10^6^ cells/mL showed higher cell viability with increases of +60% and +65% at three and seven days, respectively, as compared with the lowest dose of printed cells showing an increase of +18% at three days. For this reason, this concentration was selected for the following experiments. During culture, cell metabolic activity decreased in the NFC/A constructs at any seeded density. Moreover, at the later time point, some ASCs were adherent to the bottom surface of the well plates, suggesting their migration out of the construct.

**Figure 5:**
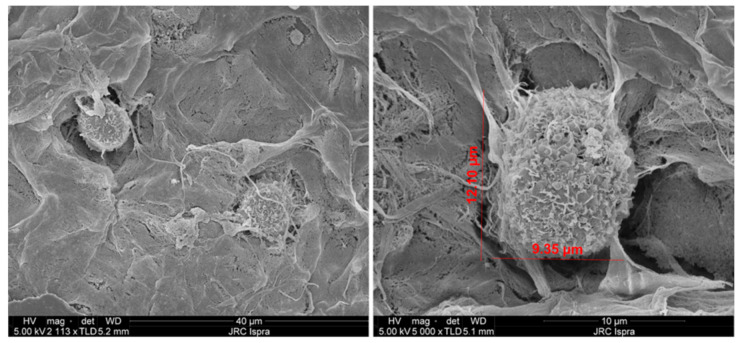
ASC appearance on the NFC/A 3D scaffold surface seeded with 6.0 × 10^6^ ASCs/mL and culture for 14 days. Micrographs obtained by SEM showing the interaction of ASCs by cytoskeleton filaments with the surrounding enabling cell–cell contacts.

In order to study the capability of the NFC/A hydrogel to hold an ASC 3D cell culture, the morphological cell appearance on the nanostructure of the scaffold was evaluated by SEM. As reported in Figure 5, 14 days after bioprinting of the cell-embedding bioink at 6.0 × 10^6^ ASCs/mL, cells appeared with a rough surface texture and were able to interact with the surrounding area and neighboring cells through their own cytoskeleton filament elongations.

### 2.4. Tenogenic Potential of the 3D ASC NFC/A Constructs

**Figure 6:**
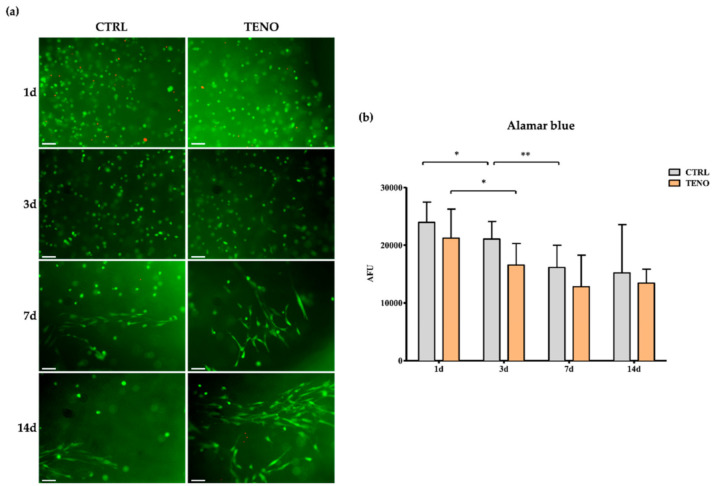
Cell viability: (**a**) representative images of live (green) and dead (red) cells encapsulated in the scaffolds at a cell density of 6.0 × 10^6^ cells/mL and cultured in undifferentiated (CTRL) and differentiated (TENO) medium after 1, 3, 7 and 14 days of cell culture (10× magnification; scale bar 10µm: N = 3); (**b**) cell viability of encapsulated ASCs at 1, 3, 7 and 14 days of cell culture (*N* = 3). Data were expressed as average ± standard deviation of arbitrary fluorescence units (AFU). Level of significance: ** *p* < 0.01, * *p* < 0.05.

In order to study the tenogenic differentiative capability of 3D-bioprinted ASCs within the NFC/A matrix, TENO serum-free culture media were supplied to the 3D-printed constructs the day after being bioprinted and the cell viability, morphology and tendon-related protein expression were evaluated over time. First, cell viability in terms of survival and metabolic/proliferation activity of both undifferentiated (CTRL) and differentiated (TENO) 3D-bioprinted ASC constructs was evaluated at 1, 3, 7 and 14 days using both the fluorescence-based live and dead and the Alamar blue assays (Figure 6). At all time points, most of the entrapped cells (over 90%) successfully survived in both CTRL and TENO conditions (Figure 6a). However, as shown in Figure 6b, cell metabolic activity progressively decreased over time up to seven days, without any significant difference between CTRL- and TENO-cultured ASCs. In particular, CTRL-cultured constructs showed a significant decrease in cell viability of 12.1% (*p* < 0.05) and 32.6% (*p* < 0.01) after three and seven days, respectively, compared to cell viability at day 1. Similarly, decreases of 22.0% after three days (*p* < 0.05) and 46.6% after seven days were observed in TENO-cultured constructs with respect to day 1. Finally, at 7 and 14 days of culture, no further cell viability decreases were observed. Interestingly, at these time points, 3D-printed ASCs exerted an evident cell spreading and orientation towards a preferred direction, irrespective of the culture medium, probably induced by the bioprinting process (Figure 6a).

**Figure 7:**
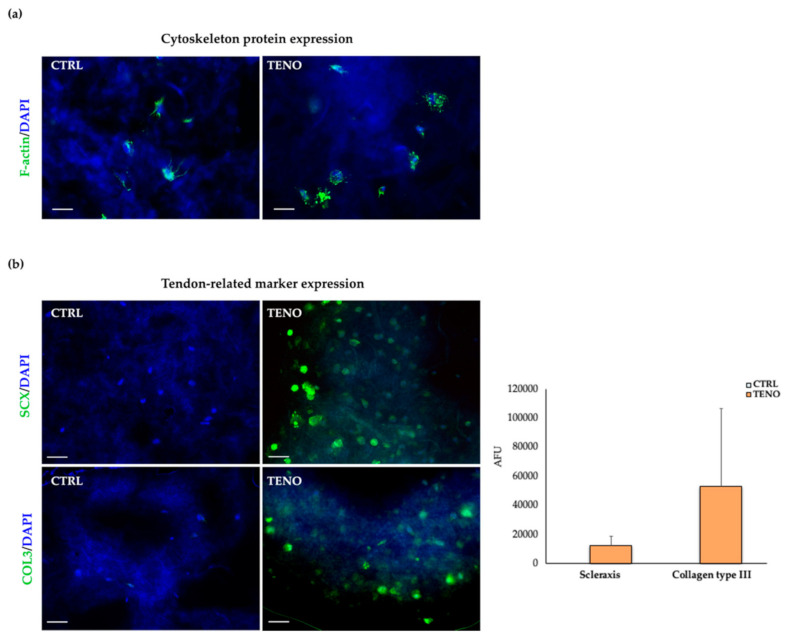
Cytoskeleton- and tendon-related marker expression: (**a**) representative images of both CTRL- and TENO-cultured ASCs embedded in NFC/A scaffolds stained at day 14 of culture with phalloidin (green) for F-actin detection and counterstained with DAPI to visualize cell nuclei (blue) (32× magnifications; 10µm scale bar); (**b**) on the left, representative images of both CTRL- and TENO-cultured ASC constructs stained with scleraxis (SCX) and collagen type III (COL3) at days 3 and 14 of differentiation, respectively, visualized with Alexa Fluor 488 (green) and counterstained with DAPI (blue) (20× magnification; 10µm scale bar); on the right, the level of cellular fluorescence intensity from fluorescence microscopy images (*N* = 3) expressed as average ± sem of arbitrary fluorescence units (AFU).

The visualization of actin cytoskeleton expression by ASCs embedded in the NFC/A matrix partially confirmed the spreading activity of cells (Figure 7a). Indeed, some actin filament extensions that spread in the 3D environment of the scaffold were observed at 14 days of culture. Interestingly, TENO-cultured ASCs seemed to exhibit a more pronounced expression of actin filaments that appeared in greater number on cell surfaces in comparison to what was observed in undifferentiated constructs (CTRL-cultured ASCs). Immunofluorescence analysis was performed to assess the presence of the tendon-related markers scleraxis and collagen type III at days 3 and 14 of differentiation, respectively. As shown in Figure 7b, TENO-cultured ASCs specifically expressed the early tendon marker scleraxis and the ECM marker collagen type III. The effective strong tenogenic differentiation was confirmed by the significant higher increases in cellular fluorescence quantified in TENO-cultured ASC constructs in comparison to CTRL-cultured ASCs that resulted as negative for both markers. These findings are in agreement with our previous in vitro study that demonstrated significant increases in scleraxis expression and ECM production after tenogenic differentiation of ASCs cultured in 2D conditions [5].

### 2.5. Inflammatory Response

**Figure 8:**
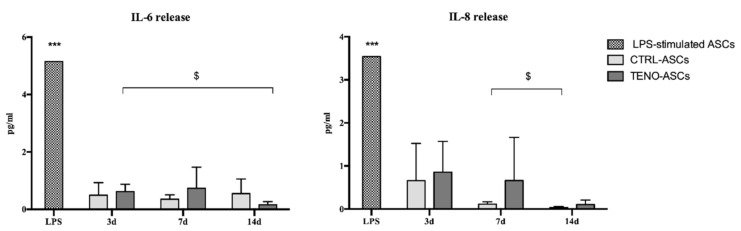
ASC cytokines release in the 3D NFC/A cultures. IL-6 and IL-8 release obtained by the supernatant media collected after 3, 7 and 14 days of both undifferentiated (CTRL) and differentiated (TENO) ASC constructs (*N* = 3). Positive control was obtained from supernatant media of ASC constructs treated with LPS for 24 h. Level of significance *** *p* < 0.001 vs. the respective no-LPS-stimulated ASC constructs, ^$^ *p* < 0.05 vs. time points.

In order to control the release of pro-inflammatory cytokines in both CTRL- and TENO-cultured ASC secretomes, cell supernatants were collected at day 3, 7 and 14 of culture and the content of GM-CSF, IFN-γ, IL-2, IL-4, IL-6, IL-8, IL-10 and TNF-α was analyzed. The supernatant of cell constructs treated for 24 h with lipopolysaccharide (LPS) was used as positive control. Neither GM-CSF, IFN-γ, IL-2, IL-4, IL-10 nor TNF-α cytokines were detected in the conditioned medium from undifferentiated and differentiated ASC constructs. On the other hand, as shown in Figure 8, increases in IL-6 and IL-8 were observed at all time points in both CTRL- and TENO-cultured ASC constructs with no differences ascribable to the culture conditions. However, the maximal mean concentration of these secreted cytokines reached levels lower than 1 pg/mL, being significantly lower than what was observed in the respective LPS-treated group (*p* < 0.001).

## 3. Discussion

The main aim of this study was to propose a tendon tissue engineering strategy through the biofabrication of a 3D cell construct containing ASCs into an NFC/A hydrogel. The proposed approach aims at recreating a suitable 3D environment in vitro, which is able to mimic the physiological cues in natural tendon tissue, and at studying its tenogenic potential by combining it with a cocktail of biochemical stimuli. We obtained successful and reliable bioprinting results such as high printing resolution and fidelity, a homogeneous cell distribution and high cell viability post-printing [42]. Various factors such as good mechanical, biodegradable and biocompatible properties of biomaterials, as well as appropriate viscosity, surface tension and crosslinking kinetics, are important to determine the printing accuracy and cell loading capacity of a bioink. A proper balance between physico-mechanical and biological properties of the selected biomaterials is needed to ensure high cell viability, differentiation and migration of seeded cells, as well as nutrient and oxygen diffusion through the bioprinted construct [43]. The NFC/A bioink studied in this work exhibited a shear thinning behavior, which is a key feature for printable formulations, with viscoelastic properties that allowed a good structural integrity after printing [32,39,43]. The hydrogel showed a high hydrophilicity after bioprinting, indicating the scaffold’s capability to absorb body fluids and diffuse cell nutrients and metabolites important to sustain cell viability and proliferation. Moreover, during the entire time in culture, the bioprinted NFC/A hydrogel showed macroscopic integrity suggesting a prolonged stability of the samples in aqueous media. The successful crosslinking of alginate confirmed by swelling/stability data on day 1 (in the absence of alginate crosslinking, the samples would solubilize very quickly) further supports the prolonged structural stability of the NFC/A scaffold. In addition, according to the supplier’s protocols, samples were incubated for characterization and cell culture in fluid media containing calcium to ensure prolonged stability in a watery environment. In accordance with our results, the recently published paper by Schmidt et al. qualitatively tested the stability of 3D-printed structures based on a similar alginate and nanocellulose-based bioink, without laminin [44]. The authors showed that 3D-printed structures starting from this bioink did not show any macroscopically visible changes, suggesting stability of the printed structures up to 14 days and the absence of significant swelling, as demonstrated by the lack of a clear increase in scaffold volume. The absence of visible swelling can be correlated to the high water content of the formulation and is in agreement with our data at day 1 which showed a retained wet weight of the samples, excluding relevant fluid absorption.

Bioink printability and cell viability are both crucial parameters for the successful bioprinting and development of specific tissue substitutes. A suitable viscosity of the gel construct is required to achieve printing fidelity as well as high cell viability post-printing [45]. Both mechanical properties of the biomaterials and the initial cell density employed for bioprinting can influence the final viscosity of the bioink [46,47]. For this reason, in order to find the optimal cell concentration within the bioink, it is essential to guarantee the final construct’s fidelity and the functionality in terms of cell survival after printing, proliferation and differentiation [48]. However, only a few studies have examined the effect of the cell seeding density in 3D cell–hydrogel systems. Krontiras et al. tested two different concentrations of mouse MSCs, i.e., 5.0 or 8.3 × 10^6^ cells/mL, in the NFC/A scaffold and did not report any influence on the cell distribution assessed by SEM [49]. Moreover, cell density can vary according to the tissue. The optimal value for bone tissue engineering has been estimated between 5 and 10 × 10^6^ cells/mL, whilst no reports concerning tendon tissue [50,51,52] are present to date, to our knowledge. Since no previous data are available for tendon tissue engineering, in this study, we investigated, for the first time, the influence of the cell density of ASCs in an NFC/A hydrogel on cell survival and proliferation. We showed a high survival rate and high viability of ASCs after printing without significant differences among 3, 6 and 9.0 × 10^6^ cells/mL of seeding density. These data also clearly demonstrated the biocompatibility of the bioink, which turned out to be a well-suited hydrogel for the 3D bioprinting of ASCs. These results are also in line with previous published studies, which reported high cell viability and proliferation post-printing of chondrocytes and induced pluripotent stem cells embedded into an NFC/A bioink [33,34,35]. Moreover, we showed that high cell viability was maintained during the time in culture with only a few dead cells detected. On the other hand, irrespective of the cell density within the initial bioink, we observed decreases in cell metabolic activity over time. According to our results, Novikova et al. reported that MSCs from bone marrow cultured in an alginate hydrogel appeared as atypical cells with spherical shape and inhibited metabolic activity [53]. Other studies reported that NFC/A hydrogels do not allow or inhibit proliferation and growth of several different types of cells [35,44]. For instance, Müller et al. also reported a significant decrease in cell proliferation of chondrocytes embedded in an NFC/A hydrogel [35]. The cellular matrix mechanics can also regulate the behavior and functionality of MSCs in terms of cellular adhesion, proliferation and differentiation. For this reason, we hypothesized that the matrix characteristics such as pore size or pore interconnectivity could not allow a proper cell metabolic activity and proliferation of ASCs in the NFC/A construct during culture. The importance of the pore size of scaffolds to ensure cell bio-adhesion, proliferation and differentiation has recently been recognized. In some reports, it was indicated that the gel pore size must be sufficiently small to allow mechanical integrity, but enough void space should be available to accomplish cell growth and nutrient diffusion within tissue constructs [54,55]. Moreover, in a recent study, it was demonstrated that the anisotropic collagen glycosaminoglycan scaffold geometry could play a key role in the promotion of tenocyte alignment along the axis of ellipsoidal pores [56]. Interestingly, the same group observed that higher crosslinking densities and smaller pore sizes were able to promote increased tenocyte metabolic activity and increase the expression of tenogenic gene expression profiles [57]. This effect could explain the negative trend of ASC proliferation observed in our NFC/A hydrogel during the time in culture. However, further investigations on ASC proliferation and tenogenic differentiation on different matrix mechanics are still needed to establish effective regenerative strategies. Further research to better control porosity could provide more insight about nutrient transport and cellular metabolic activity in long-term cultures. On the other hand, future studies with a lower cell density (<1.0 × 10^6^ cells/mL) could be also conducted to investigate if the major oxygen and nutrient availability within the constructs allows higher cell growth.

Since we did not observe a clear dependence of cell viability on the initial cell concentration within the bioink, the concentration of 6.0 × 10^6^ ASCs/mL was selected for the tenogenic potential evaluation. Interestingly, our results by SEM and actin cytoskeleton images demonstrated that the embedded ASCs were able to develop cell–cell and cell–3D environment interactions by their own cell extensions at 14 days of culture. This spreading activity was also evident in live and dead images in both CTRL- and TENO-cultured ASCs at later time points (Figure 6), though it was lacking in the images concerning the live and dead of CTRL-cultured ASCs at several cell densities (Figure 4). One explanation for this different behavior in cell extensions could be related to a non-homogeneous matrix characteristic in the final 3D construct as a result of slight variations in the bioink temperature, humidity in the nozzle or cell distribution within the bioink during the printing process of all 3D constructs that can last several minutes. The observed spreading activity of cells could exert a central role on cellular communication and tissue homeostasis. The actin cytoskeleton effect in cells’ contractile machinery and elastic modulus during early tendon development has recently been demonstrated [58]. In detail, the actin cytoskeleton is strictly involved in the typical crimp collagen pattern formation of tendon ECM and in the mechanical properties of mature tendon tissue acting both as mechano-transducer and signaling actor [59,60,61]. The contact sites of cells to ECM, the focal adhesions, form through transmembrane proteins called integrins, allowing specific recognition of various ECM proteins including collagen and laminin [62]. For these reasons, laminin proteins present within the NFC/A bioink could exert an important role in supporting cell adhesion, proliferation and differentiation. Other reports demonstrated also the key role of matrix stiffness in the modulation of tendon-derived stem cells’ (TDSCs) proliferation and differentiation [63]. In particular, phosphorylation of kinases involved after the ligation of integrins at the focal adhesion, e.g., FAK and ERK1/2, was enhanced in TDSCs cultured on stiff gelatin hydrogels and inhibited by matrices exhibiting lower stiffness. The phenomenon was especially obvious on polystyrene culture plates and in accordance with the ASC behavior, which showed a faster growth rate on stiff substrates compared to soft ones [64]. Nevertheless, it has recently been reported that stem cell types derived from soft tissues such as umbilical cord stem cells lose their cell proliferation ability if the stiffness of the matrix increases [65].

Tenogenic differentiation of stem cells, and thus the regenerative ability of tendons, is based on the combination of matrix stiffness, mechanical forces and biochemical inducing signals. Currently, there is no established protocol with endogenous inducer factors to differentiate MSCs into a tenocyte-like phenotype. Another difficulty in the study of tendon differentiation is represented by the limited number of tendon markers present to date. The main structural and functional component of the tendon matrix consists of collagen type I, followed by collagen type III, but it is not specific and is expressed in several tissues [66]. To date, the bHLH transcription factor scleraxis is the best recognized marker of tendon tissue development [8,67]. In this study, we demonstrated, for the first time, that TENO-induced bioprinted ASCs in the NFC/A bioink specifically expressed tendon-related markers of the early and late events of tendon development, namely scleraxis and collagen type III, respectively. Accordingly, we have recently reported that a combination of BMP-12, CTGF, TGF-β3 and ascorbic acid in a serum- and xenogenic-free medium was able to efficiently drive the tenogenic differentiation of ASCs cultured on collagen type I-coated flasks [5]. The biochemical stimulation of ASCs induced a strong and specific expression of scleraxis and collagen type I and III at a significantly higher level in comparison to CTRL cells. Moreover, the NFC/A hydrogel did not exert any inflammatory response to ASCs.

Our approach, based on the choice of commercially available FDA-approved materials, i.e., NFC/A, and of our in-house GMP-compliant tenogenic protocol, represents an important step in moving forward the clinical application of the 3D-bioprinted product. These results represent, to our knowledge, the first attempt to investigate the influence of an NFC/A hydrogel and tenogenic medium on the cytokines release of ASCs. Undifferentiated ASCs secrete a broad panel of growth factors and cytokines with trophic, immunomodulatory, antiapoptotic and proangiogenic properties. Further investigations on the complete panel of cytokines released from ASCs after bioprinting could provide additional insight into their biological response. However, considering the mechano-responsiveness nature of tendon tissue, and in order to better reproduce the natural tendon microenvironment, future investigations may be performed by dynamic cultures using a bioreactor, elucidating the mechanical response of the 3D ASC constructs in terms of tenogenic differentiative ability. Moreover, preclinical investigations in animal models are required to better address the safety and efficacy of the 3D ASC constructs in terms of the biocompatibility and degradability of the scaffold and in ameliorating and promoting the regeneration of tendon tissue.

## 4. Materials and Methods

### 4.1. 3D Printing of Nanocellulose–Alginate Scaffolds

**Figure 9:**
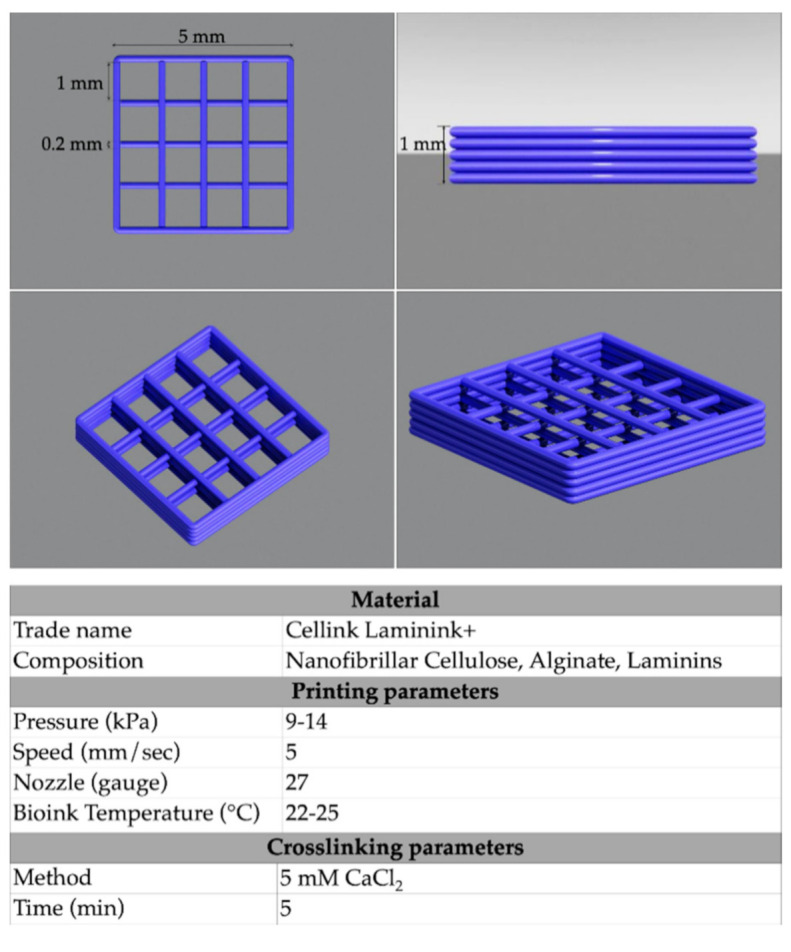
3D scaffold design: upper panels show top (**left**) and side (**right**) views, bottom panels show perspective projection images of the 3D NFC/A scaffold. The table reports the materials and methods used for the 3D bioprinting of ASCs in the NFC/A hydrogel.

In this study, a micro-extrusion-based three-syringe cell bioprinter (Biox, Cellink^®^, Boston, MA, USA) was used to print the NFC/A scaffolds. The bioink (Laminink+, Cellink^®^, Boston, MA, USA) was a mixture of nanofibrillar cellulose (NFC; CAS 9004-34-6, Sigma-Aldrich, St. Louis, MO, USA) and sodium alginate (A; CAS 9005-38-3, Sigma-Aldrich) with the addition of ECM proteins laminin 111, 121, 411 and 521. The scaffold design model used was a square of 5 × 5 × 1 mm dimension with a 200 µm layer height and nozzle diameter and 20% infill density determined by the G-Code file of the printing device software (Figure 9). The resulting projected area of each scaffold was 5 × 5 mm and scaffolds consisted of 5 layers. In the Figure 9 the detailed printing and crosslinking parameters were also listed.

To repeat the same methodology used to bioprint cells, the injectable ink was prepared by mixing 1 mL of hydrogel with 100 μL of Minimum Essential Medium (MEM)α without nucleosides with Glutamax or Hanks’ Balanced Salt (HBSS^+/+^) solution with calcium and magnesium (both by Thermofisher Scientific, USA). For the printing procedure, the prepared ink was introduced into a sterilized 3 mL polyethylene injection cartridge (Cellink^®^) and then fixed into the printing device previously sterilized with UV light. After G-Code model selection, scaffolds were printed layer-by-layer via ink extrusion as a fiber in a 24-multi-well plate at room temperature (about 25 °C). Typically, air pressure was set to 9–14 kPa, speed was 5 mm/s and nozzle size was fixed at 27 gauges. Nanocellulose–alginate scaffolds were immersed in 5 mM CaCl_2_ solution (Cellink^®^) for 5 min to crosslink alginate, thus stabilizing the porous structure. Then, scaffolds were washed three times with HBSS^+/+^ solution to remove residual CaCl_2_..

### 4.2. Rheological Characterization of the Bioink

Rheological characterization of the NFC/A bioink was conducted using a stress-controlled rheometer (MCR302 Anton Paar, Graz, Austria) equipped with a Peltier system for temperature control and a 25 mm parallel-plate geometry. In detail, the native bioink was characterized before crosslinking by rheological strain sweep and frequency sweep tests at 25 °C, that is, the cartridge and printing platform temperature during the layer-by-layer deposition. In detail, the strain sweep test was conducted at constant frequency (1 Hz) within the strain range from 0.01 and 500% with the aim to define the linear viscoelastic (LVE) region and yield stress (YS, i.e., the value of shear stress at the maximum of the loss modulus) that characterized the bioink. Then, the frequency sweep test was conducted within the LVE by varying the angular frequency within the range 0.1–100 rad/s. The same characterizations were also conducted on the crosslinked bioink to assess the effects of the crosslinking process on the rheological properties of the gel. The crosslinked gel was also characterized at 37 °C, that is, the temperature in the incubator during 3D cell cultures. Before each test, the bioink was deposited on the bottom plate of the rheometer at 25 °C and left to equilibrate for 10 min at the testing temperature before the analysis. In order to test the crosslinked bioink, the hydrogel was crosslinked for 5 min according to the supplier’s instructions and then equilibrated at the testing temperature for 10 min and finally tested.

### 4.3. Scanning Electron Microscopy (SEM)

The porosity and ultrastructural morphology of fabricated scaffolds were assessed by scanning electron microscopy (Thermofisher-FEI Nova NanoLab 600 DualBeam). Samples were dehydrated in 30, 50, 75, 95 and 100% ethanol solutions and then transferred to the critical point dryer (K850 Quantum Design GmbH, Darmstadt, Germany). Dried scaffolds were mounted on self-adhesive carbon disks and sputter-coated with a conductive thin layer of gold using a magnetron sputtering reactor equipped with a pure Au target and operated in DC mode with a bias voltage of 150 V (Leybold, Cologne, Germany). The microscope was driven with an acceleration voltage of 5.0 kV detecting secondary electrons, a working distance (WD) of 5 mm and a spot current of 98 pA, using 32×, 300×, 1000× and 80,000× magnification scales.

Cell distribution in the scaffolds was visualized by SEM after 14 days of culture. To this aim, samples were fixed in 4% formaldehyde (Sigma Aldrich, St. Louis, MO, USA) for 1 h at RT. Dried samples were obtained by dehydration in ethanol gradient series followed by critical point drying and then visualized as above. The microscope was driven as before and using 113×, 5000× and 8000× magnification scales.

### 4.4. Swelling Ratio

The fluid content of the prepared hydrogel scaffolds (*N* = 6) was measured immediately after gelation (0 h) and after 24-h incubation in culture medium (500 μL/scaffold). At each time point, scaffolds were slightly dried with a filter paper to remove surplus aqueous medium and weighed (*Ws*). Then, samples were dried at 50 °C in a dryer for 30′ and weighed again (*Wd)*. The swelling capacity of the hydrogels was estimated using the equation

$$\mathrm{Swelling}\%=\frac{\left(Ws-Wd\right)}{Wd}\times 100$$

### 4.5. Cell Isolation and Culture Expansion

Human adipose-derived stem cells were isolated from subcutaneous adipose tissue of 4 healthy donors undergoing liposuction and their full characterization in terms of surface antigen expression, morphology and differentiative capability was already demonstrated and reported elsewhere [5]. Importantly, in this study, we used the same ASC population characterized before in 2D in vitro conditions. All the medical procedures were approved by the Ethical Committee of the Canton Ticino, Switzerland (CE 2961). All subjects were in good health and provided their written consent before participating in the study. The collected stromal vascular fractions were plated at a cell density of 10^4^ cells/cm^2^ and cultured in a 5% human platelet lysate xenogenic-free expansion medium consisting of MEMα without nucleosides with Glutamax (Fisher Scientific, Hampton, NH, USA) supplemented with 5% pooled human platelet lysate (hPL, Stemulate, Cook Regentec, Indianapolis, IN, USA), 100 Units/mL of Penicillin and 100 Units/mL of Streptomycin (Fisher Scientific, USA). The medium was changed every three to four days and cells were passaged upon reaching 90%. After approximately 7 days of culture, cells were harvested by trypsin treatment (0.5% trypsin/0.2% disodium ethylenediaminetetraacetic acid, EDTA; Sigma-Aldrich, USA) for 5 min and then counted by trypan blue exclusion. To obtain the number of cells needed for the 3D bioprinting experiments, cells were plated on T150 cell culture flasks (Nunc) at a cell density of 3000 cell/cm^2^ and cultured in the 5% hPL xenogenic-free expansion medium. For cryopreservation, cells were centrifuged at 400 g for 5 min, resuspended in an ice-cold solution of 1% albumin solution, 5.5% ME_2_SO and 4.5% dextran-40 (Cryosure DEX-40, WAK-Chemie Medical GmbH, Steinbach, Germany) in MEMα and transferred into a 2 mL cryovial (Nalgene, Thermo Fisher Scientific, Waltham, MA, USA). As previously described, cells were frozen by means of a programmable freezer (Consartic GmbH, Westerngrund, Germany) [5]. Cryovials were then transferred into liquid nitrogen for long-term storage. After thawing, cells at passage 3 (*N* = 4) were analyzed for mycoplasma detection. Samples were prepared by collecting 5.0 × 10^5^ ASCs and their supernatant of three days of culture, which were then analyzed by Mycoplasma Diagnostic Service (Minerva Analytix GmbH, Rangsdorf, Germany). All samples resulted as negative for mycoplasma contamination. For the 3D bioprinting experiments, ASC populations at passage 4 were used.

### 4.6. Bioprinting of ASCs

For the bioprinting of cellularized constructs, ASCs at passage 4 (*N* = 3) were resuspended within the bioink (Laminink+) at a final concentration of 3, 6 or 9 × 10^6^ of ASCs per mL of bioink (*N* = 3) to test the effect of different cell densities on cell viability and morphology after printing. To this aim, pellets were resuspended in 100 µL of CTRL medium consisting of MEMα without nucleosides with Glutamax (Fisher Scientific) supplemented with 2% pooled human platelet lysate (hPL, Stemulate, Cook Regentec, USA) and 100 Units/mL of Penicillin and Streptomycin (Fisher Scientific). Afterwards, ASC suspensions were gently mixed with 1 mL of bioink at room temperature (about 25 °C) through sterile luer locked syringes; the resulting cellularized bioink was then dispensed in an empty cartridge and finally fixed into the printing device. After scaffold design selection, cell-laden scaffolds were printed layer-by-layer in 24-multi-well plates. The ratio between material and cells, as well as the nozzle diameter, was kept constant, and the printing pressure was adjusted as required in the range of 9–14 kPa (Figure 9). After crosslinking, the cell-laden scaffolds were cultured for 14 days in CTRL medium consisting of MEMα without nucleosides with Glutamax (Fisher Scientific, USA) supplemented with 2% pooled human platelet lysate (hPL, Stemulate, Cook Regentec, USA), 100 Units/mL Penicillin and 100 Units/mL of Streptomycin (Fisher Scientific, USA) at 37 °C 5% CO_2_ in a humidified incubator.

### 4.7. Cell Viability

A fluorescence-based live and dead assay was used to evaluate cell viability in the bioprinted 3D cell-laden scaffolds at 1, 3, 7 and 14 days in all culture conditions (N = 3). The simultaneous use of two fluorescent dyes allowed a two-color discrimination of the population of living cells from the dead cell population. In particular, after washing with HBSS twice, samples were stained using 20 mg/mL fluorescein diacetate (FDA) and 1.6 mg/mL propidium iodide (PI) (both from Sigma-Aldrich) in MEMα, for 5 min at room temperature (RT) in the dark. Viable cells were visualized as green fluorescence under the Green Fluorescent Protein (GFP) channel (emission: 525 nm), whereas dead cells were visualized under the mCherry Fluorescent Protein channel (emission: 630 nm). A fluorescent microscopy Zeiss Axiovert Apotome was used to visualize the living and dead cells and three representative images were taken from each scaffold (magnification of 10×).

Quantitative analysis of cell viability and proliferation was performed in all 3D culture conditions by Alamar Blue Assay (Thermo Fisher Scientific, Waltham, MA, USA) at 1, 3, 7 and 14 days of culture after printing [68]. At the day of the evaluation, cells were incubated with Alamar Blue (1:10 dilution in MEMα) for 4 h at 37 °C in the dark. Then, supernatants were transferred to black-bottom 96-well plates and emitted fluorescence was read with an Enspire plate reader (Perkin Elmer, Waltham, MA, USA).

### 4.8. Tenogenic Differentiation

For tenogenic differentiation evaluation, the cell-loaded hydrogel at 6.0 × 10^6^ ASC/mL cell density was used and 3D-printed scaffolds were cultured in CTRL medium at 37 °C 5% CO_2_ as described before (N = 3). The day after, tenogenic differentiation was induced as described before by culturing ASC-embedding scaffolds in TENO medium consisting of CTRL medium supplemented with 50 μg/mL ascorbic acid (AA; Sigma Aldrich), 50 ng/mL BMP-12, 100 ng/mL CTGF and 10 ng/mL TGF-β3 (all from PeptroTech, Rocky Hill, NJ, UK) [5]. Differentiation was induced up to 14 days of culture, changing the medium twice a week. Cells cultured in CTRL medium without any further supplementation were also used as control.

### 4.9. Immunofluorescence Staining

Cell morphology appearance in both CTRL- and TENO-cultured 3D cell-laden constructs was evaluated after 14 days of cell culture by assessing the distribution of F-actin in fixed cells through the use of fluorescently conjugated phalloidin (N = 3). Cell constructs were fixed with 4% paraformaldehyde in HBSS, then washed three times with HBSS and permeabilized with 0.5% Triton X-100 in HBSS (HBSST). After three washes with HBSS, samples were stained with 1:100 DAPI (Invitrogen) for 15 min at RT in the dark and then washed as before. Constructs were stained for 20 min at 37 °C in the dark using 1:40 Alexa Fluor 488 phalloidin antibody (Invitrogen, Thermo Fisher Scientific), then washed three times with HBSS and prepared for cryo-section. Samples were embedded in tissue-freezing medium (Leica, Weitzlar, Germany) and then frozen at −20 °C. The frozen samples were transversally cryo-sectioned along microgrooves to 50 µm sections using a cryostat (Leica CM 1800). Six sections for each sample were observed and photographed under a fluorescence microscope (Zeiss Axiovert Apotome).

Expression of the transcription factor scleraxis and the extracellular matrix protein collagen type III was assessed by immunofluorescence staining after 14 days of culture in both CTRL and TENO cell culture medium (*N* = 3). Cells in the constructs were fixed with 4% paraformaldehyde in HBSS, then washed three times with HBSS, permeabilized with 0.5% Triton X-100 in HBSS (HBSST) and blocked with bovine serum albumin (BSA; from Sigma Aldrich). Immunostaining was performed overnight at 4 °C using 1:100 goat anti-human Scleraxis (HPA043183, Sigma Aldrich) and 1:500 rabbit anti-human Collagen III (PA5-34787, Invitrogen). Cells were washed three times in HBSST, incubated for 1 h at room temperature with 1:1000 Alexa Fluor 488 rabbit anti-goat IgG (Invitrogen, Carlsbad, CA, USA) and cell nuclei were counterstained with 1:100 DAPI (Invitrogen). Immunostained constructs were cryo-sectioned as described before and six 50 µm sections for each sample were observed and photographed under a fluorescence microscope (Zeiss Axiovert Apotome). The level of cellular fluorescence from fluorescence microscopy images (*N* = 3) was determined by ImageJ software and expressed as average ± sem of arbitrary fluorescence units (AFU).

### 4.10. Cytokines Release

To identify biomarkers of inflammation, GM-CSF, IFN-γ, IL-2, IL-4, IL-6, IL-8, IL-10 and TNF-α were analyzed by measuring the secreted amount in the supernatant of both CTRL- and TENO-cultured cell-laden constructs after 3, 7 and 14 days of culture (*N* = 3). The conditioned medium of samples treated with 20 ng/mL LPS (Lipopolysaccharides; Invitrogen) for 24 h was used as positive control.

Cytokine release was tested through a multiplex enzyme-linked immunosorbent assay (ELISA) by using a Bio-Plex Pro Human Cytokine 8-plex assay. Each experimental sample was run in a biological duplicate. Cytokines were measured with a Bio-Plex200 System using the Bio-Plex ManagerTM software. All reagents and instruments including the Wash Station and Shaking Incubator were from BIORAD (Hercules, CA, USA).

### 4.11. Statistical Analysis

Data were expressed as means ± standard deviations (sd) or mean ± standard error mean (sem) The normal distribution of values was assessed by the Kolmogorov–Smirnov normality test. Statistical analyses were performed using the Student’s t-test for data with a normal distribution and the Wilcoxon test for data with a non-normal distribution (GraphPad Prism v6.00; GraphPad Software, USA).

## 5. Conclusions

Tissue engineering has recently been proposed to improve natural tendon healing as an alternative strategy to unsuccessful current conventional treatments. A key challenge is the definition of the best combination of cells and biomaterials; cells have a primary role in maintaining tendon ECM homeostasis, while biomaterials should possess certain requirements in terms of mechanical, structural and biological properties as they could also modulate cell proliferation and differentiation. Most importantly, several key issues must be considered, such as the lack of standardized protocols defining the resolution of the printing process, the definition of adequate differentiation protocols of MSCs and their bioactivity in cell constructs. Moreover, despite the growing knowledge on stem cell biology and regeneration and their safety and effective use in cell-based therapies for several diseases, stem cell therapies for tendons require further research to better understand the mechanisms of the tenogenic process and associated markers, together with the translation of appropriate stimuli in a spatial-temporal manner toward successful cell-based therapeutic tools.

To address these challenges, we showed that the 3D bioprinting of ASCs into an NFC/A hydrogel could provide a xenogeneic-free tool that might be useful in improving healing in tendons, thus opening new possibilities in personalized medicine. In fact, for the first time, we evaluated ASCs’ behavior after their embedding into the NFC/A hydrogel and bioprinting into square-grid structures, in terms of cell viability, proliferation, differentiation and inflammatory response. The influence of the cell seeding density into the bioink on cell behavior upon printing was also assessed. Primary human adipose-derived stem cells showed a high survival rate after printing and cell viability in 3D culture was not influenced by cell seeding density in the starting bioink, demonstrating its potential as a cell-carrier for the fabrication of 3D tissue constructs. However, cell viability decreases were observed after seven days of culture in both CTRL- and TENO-cultured ASCs.

Finally, in view of future in vivo applications, with this study, we obtained a good biological response of human ASC cellular viability, tenogenic potential and immunomodulatory properties in the NFC/A hydrogel. In fact, the presence of biochemical and physical stimuli appeared to have an influence on cell differentiation toward the tendon lineage, as demonstrated by the specific and strong protein expression of the early marker scleraxis and the late marker collagen type III. Moreover, the absence of an ASC inflammatory response to the NFC/A substrate represents another insight that ensures the safety of these xeno-free FDA-approved materials and of the xenogenic-free tenogenic differentiation protocol.

In conclusion, all these findings represent the first proof of concept for the possible development of ASC-laden 3D-bioprinted constructs using FDA-approved materials and clinical-grade reagents needed for cell-based therapy and tissue engineering applications. However, critical challenges still remain to be addressed in order to apply 3D bioproducts in clinical settings, such as construct fabrication, standardized markers for cell viability and functionality in the 3D construct and standardization of bioprinting parameters. The way towards ad hoc cell-laden implant development is in its infancy and further research to demonstrate the in vivo functionality is needed.

## Abbreviations

ASCs	Adipose-derived stem cells
MSCs	Mesenchymal stem cells
TE	Tissue engineering
GMP	Good manufacturing practice
ECM	Extracellular matrix
3D	Three-dimensional
NFC	Nanofibrillar cellulose
A	Alginate
SEM	Scanning electron microscopy
BMP-12	Bone morphogenic protein-12
TGF-β3	Transforming growth factor-beta3
CTGF	Connective tissue growth factor
ILs	Interleukins
FBS	Fetal bovine serum
LVE	Linear viscoelastic region
YS	Yield stress
SCX	Scleraxis
COL3	Collagen type III
LPS	Lipopolysaccharide
